# Practical Guidelines on Implementing Hypofractionated Radiotherapy for Prostate Cancer in Africa

**DOI:** 10.3389/fonc.2021.725103

**Published:** 2021-12-01

**Authors:** William Swanson, Richard Ndi Samba, Michael Lavelle, Ahmed Elzawawy, Erno Sajo, Wilfred Ngwa, Luca Incrocci

**Affiliations:** ^1^ Department of Physics and Applied Physics, University of Massachusetts Lowell, Lowell, MA, United States; ^2^ Department of Radiation Oncology, Dana Farber Cancer Institute, Brigham and Women’s Hospital, and Harvard Medical School, Boston, MA, United States; ^3^ Department of Regulation and Regulatory Control, Cameroon National Radiation Protection Agency, Yaounde, Cameroon; ^4^ Department of Clinical Oncology, Suez Canal University, Ismailia, Egypt; ^5^ Department of Radiation and Molecular Radiation Sciences, Johns Hopkins University, Baltimore, MD, United States; ^6^ Department of Radiotherapy, Erasmus Medical Center (MC), Rotterdam, Netherlands

**Keywords:** prostate cancer, hypofractionation, radiotherapy, guidelines, Africa

## Abstract

Among a growing body of literature in global oncology, several articles project increased cost savings and radiotherapy access by adopting hypofractionated radiotherapy (HFRT) in low- and middle-income countries (LMICs) like those in Africa. Clinical trials in Europe and the USA have demonstrated HFRT to be non-inferior to conventional radiotherapy for eligible patients with several cancers, including prostate cancer. This could be a highly recommended option to battle a severely large and growing cancer burden in resource-limited regions. However, a level of implementation research may be needed in limited resource-settings like in Africa. In this article, we present a list of evidence-based recommendations to practice HFRT on eligible prostate cancer patients. As literature on HFRT is still developing, these guidelines were compiled from review of several clinical trials and professionally accredited material with minimal resource requirements in mind. HFRT guidelines presented here include patient eligibility, prescription dose schedules, treatment planning and delivery techniques, and quality assurance procedures. The article provides recommendations for both moderately hypofractionated (2.4-3.4Gy per fraction) and ultrahypofractionated (5Gy or more per fraction) radiation therapy when administered by 3D-Conformal Radiotherapy, Intensity Modulated Radiation Therapy, or Image-Guided Radiotherapy. In each case radiation oncology health professionals must make the ultimate judgment to ensure safety as more LMIC centers adopt HFRT to combat the growing scourge of cancer.

## Introduction

In 2020, prostate cancer claimed more than 47,000 lives with over 93,000 new cases in Africa (1). The Global Cancer Observatory projects this to increase to 111,000 new incidences and 56,000 deaths in 2025 (**Supplementary Figure 1**) (1). This significant burden is currently managed by an estimated 246 geographically sparse radiotherapy (RT) centers (**Supplementary Figure 2**) serving a population of over 1.2 billion (2). Many clinical trials conducted in Europe and North America have determined recommended parameters to deliver prostate hypofractionated radiotherapy (HFRT) safely with non-inferior outcomes to conventionally-fractionated RT (CFRT) (3–5). Evidence-based HFRT for prostate cancer is characterized by the delivery of greater than 2 Gy per fraction and a reduced number of fractions as compared to conventional fractionation (6). For prostate cancer, a typical CFRT prescription would be 78 Gy in 39 fractions (five fractions per week) (7). In comparison, an equivalent HFRT prescription would be on the order of 60 Gy in 20 fractions (6).

One of the clinical trials demonstrating non-inferior outcomes when using HFRT versus CFRT for prostate cancer treatment was the CHHIP trial. The 2016-reported phase III CHHIP trial showed 5-year failure free outcomes at 91% for HFRT (60 Gy in 20 fractions) patients and 88% for CFRT (74Gy in 37 fractions) patients (5). Similarly, the 2016-reported phase III HYPRO trial demonstrated 5-year relapse-free survival of 80% and 77% for HFRT (64.6Gy in 19 fractions) and CFRT (78 Gy in 39 fractions) patients, respectively (3). Additionally, the recently reported phase III HYPO-RT-PC Ultra-HFRT demonstrated no statistically significant outcome differences between CFRT (78 Gy in 39 fractions) patients and the patients receiving the more extreme Ultra-HFRT of 42.7 Gy in only seven fractions (4).

Meanwhile, several activity-based-costing models have estimated significant reduction in treatment costs and increase in treatment access by adopting HFRT for eligible patients (7, 8). While, these studies encourage the adoption of HFRT, African low- and middle-income countries (LMICs) clinics may require additional knowledge and resources to perform HFRT safely and effectively (9). With increased dose per fraction characteristic of HFRT, comes the need for increase in safety and quality assurance (QA) requirements. Recent investigation to determine the “readiness” of a sample of African clinics to adopt HFRT showed that although most clinics are already practicing HFRT for palliative care, a greater investment in infrastructure and training may be needed to provide adequate QA for safe and effective delivery of HFRT with curative intent (9).

In this article, we highlight evidence-based recommendations for performing prostate HFRT, which can serve as uniform guidelines in African LMICs, with minimal resource requirements in mind and the recognition that additional implementation research may be needed including hypofractionation clinical trials in Africa. The recommendations in this article were compiled based using evidence-based material including the HFRT clinical trials and professional RT and QA guidelines.

## Materials and Methods

The guideline was developed based on a systematic literature search and review in MEDLINE PubMed of English-language studies published between January 1, 2017 to April 30, 2021. Both Medical Subject Headings terms and text words were used, and hand searches supplemented the electronic searches. The included randomized controlled trials (RCTs) evaluated men with localized prostate cancer receiving HFRT to the prostate. Appropriate task group reports (TGRs) by the American Association of Physicists in Medicine (AAPM) served as a basis for treatment planning and quality assurance recommendations for safe delivery of HFRT by 3D Conformal Radiotherapy (3DCRT), Intensity Modulated Radiation Therapy (IMRT) and Image Guided Radiotherapy (IGRT).

### Recommendations for HFRT Practice

#### Patient Selection

Various clinical trials and professional guidelines describe the conditions under which prostate cancer patients should be offered HFRT. A summary of these criteria are provided in **Table 1**. The American Society for Radiation Oncology (ASTRO), American Society of Clinical Oncology (ASCO), and American Urological Association (AUA) recommend offering HFRT to prostate cancer patients under the following circumstances: low-risk patients selected for external-beam RT (EBRT) who decline active surveillance, intermediate-risk patients, or pelvic node-negative high-risk pelvic patients receiving EBRT (6). Literature also supports that HFRT regimen outcomes are not impacted by age, comorbidity, anatomy, or urinary function (6). The HYPRO trial was conducted with patients 44-85 years of age with stage T1B-T4NX-N0MX-M0 localized prostate cancer, PSA less than 60μg/L, and 0-2 WHO performance status (3). CHHIP trials participants included men older than 16 years of age with stage T1B-T3A-N0-M0 prostate cancer, PSA less than 40μg/L, and 0-1 WHO performance status (5). The more extreme Ultra-HFRT HYPO-RT-PC trial recruited intermediate-to-high-risk (T1C-T3A with Gleason score > 7 or PSA = 10-20μg/L) patients up to 75 years of age with 0-2 WHO performance status (4). All referenced trials above had all cases histologically confirmed prior to inclusion, and excluded patients with previous pelvic radiotherapy or presence of pelvic nodal disease (3–5).

**Table 1:** A summary of recommended prostate cancer HFRT treatment parameters.

Subject	Recommendation
Patient Eligibility	Low-risk patients declining surveillance (6) Intermediate-risk patients (6) High-risk pelvic node-negative patients (6) PSA < 60μg/L (3–5)
Prescription Options	20 x 3Gy (D_Rx_ = 60Gy) (5, 6) 28 x 2.5Gy (D_Rx_ = 70Gy) (6) 19 x 3.4Gy (D_Rx_ = 64.6Gy) (3) 7 x 6.1Gy (D_Rx_ = 64.6Gy, Ultra-HFRT) (4)
Target Volume	GTV = CTV (4, 10) ITV = CTV + margin (if needed) (10) PTV = CTV + 3-10mm margin (3, 4) 0mm CTV margin towards rectum (3–5)
Dose Distribution	D_Rx_ defined as PTV D_mean_ (4) Rectum: V_100%_ < 3%, V_68%_ < 60% (5) Bowel: V_68%_ < 17cc (5) Bladder: V_100%_ < 5%, V_68%_ < 50% (5) Femoral Heads: V_100%_ < 70% (4), V_68%_ < 50% (5) Urethral Bulb: V_81%_ < 10%, V_68%_ < 50% (5)
Treatment Planning	CT simulation with maximum slice thickness of 3mm (4, 10) Extend CT scan 5-10cm superior/inferior to treatment fields (10) Maximum 5mm MLCs required (4, 10) D_beam_ < 30% D_cumulative_ (10)
3DCRT	Minimum 10MV beam (Ultra-HFRT) (4, 10) MU output QA required (11, 12)
IMRT	Minimum 6MV beam (Ultra-HFRT) (4, 10) Direct dose output QA per plan required (12)
IGRT	Mark target volume with surgical fiducials (4, 10) Verify prostate position each fraction using on-board/integrated kV/MV/CBCT systems (4) 2mm precision required (13)
Motion Management	Use supine/prone body frames with additional techniques as needed (4, 10) Refer to AAPM TGRs 101 and 76 for recommendations regarding motion management techniques (10, 14).

#### Prescription

It is recommended that selected prescription dose and fractionation schedules should not deviate from published material unless treatment involves a clinical trial (10). Trial-recommended moderate HFRT schedules include the following: 60Gy in 20 3 Gy-fractions, 64.6 Gy in 19 3.4 Gy-fractions, or 70 Gy in 28 2.5 Gy fractions (**Table 1**) (3, 5, 6). Additionally, Ultra-HFRT has been practiced using 42.7 Gy in 7 6.1Gy-fractions (4). As HFRT literature is relatively new, quantities such as biologically equivalent dose, normalized total dose and equivalent uniform dose may not accurately describe the biological effects from HFRT and therefore, should be considered with caution when deciding on an appropriate prescription dose (10). For contouring, the gross-target-volume (GTV) and clinical-target-volume (CTV) should match and include the entire prostate (4, 10). An internal-target-volume (ITV) expansion from the CTV may be needed for motion correction but typically the planning target volume (PTV) should be defined as a 7 mm margin expansion of the CTV (4, 10). The prescription dose should be defined as the mean PTV dose with a steep, uniform, and isotropic dose gradient (4, 10).

#### Treatment Planning

Several considerations are necessary to address RT toxicity risks (**Table 1**). There is evidence that both CFRT and HFRT have similarly small increased risk of acute and late gastrointestinal toxicity when delivered by EBRT (6). To minimize this risk, the rectum and bladder should have both a near-prescription and near-midpoint dose-volume constraint (6). Additionally, physicians should schedule a follow up similarly to existing RCTs (5-6yrs) (6). The rectum, colon, urinary bladder, femoral heads, and penile bulb should be considered as organs-at-risk (OARs) during treatment planning (4). OAR dose constraints should be defined by published material (6). The CHHIP trial utilized the following recommended dose-volume constraints for a 3 Gy/fx HFRT schedule: rectum (V_41%_ < 80%, V_100%_ < 3%), bladder (V_68%_ < 50%, V_100%_ < 5%), femoral heads (V_68%_ < 50%), bowel (V_68%_ < 17cc), urethral bulb (V_68%_ < 50%, V_81%_ < 10%) (5). To compare, the HYPO-RT-PC trial utilized the following constraints for a 6.1 Gy/fx Ultra-HFRT schedule: rectum (V_65%_ < 45%, V_95%_ < 95%), femoral heads (V_100%_ < 70%), cumulative global dose does not exceed 105% the prescribed PTV dose (4). Additional recommendations on dose-volume constraints for high-dose fraction RT can be found in TGR 101 for stereotactic body RT (SBRT) (10).

Moderate HFRT schedules may be performed using LINACs by 3DCRT or IMRT methods. IMRT LINAC delivery by IGRT is preferred (**Table 1**) (3, 5). Ultra-HFRT schedules are recommended to be delivered by a LINAC using 3DCRT with a minimum 10 MV beam or IMRT with a minimum 6 MV beam (4). Unless otherwise specified, the recommendations provided here may be applied regardless of choice between 3DCRT, IMRT, or IGRT while considering past clinical trials as reference. For conservative measures on beam parameters for shortened RT schedules, TGR 101 for SBRT should be consulted. Beams should be collimated by 5 mm multi-leaf collimators (MLCs) with no single beam contributing more than 30% of the cumulative dose (10). 3mm MLCs have shown negligible advantage for lesions larger than 3 cm in diameter (10).

#### Treatment Delivery

Computed tomography (CT) treatment simulation is mandatory for basic positioning and registration (4, 10). CT should be performed, covering 5-10 cm superior and inferior beyond the treatment field, with a slice thickness of 3 mm or less (4, 10). The prostate’s position should be verified by a kV or MV electronic portal imaging device (EPID), or cone-beam CT (CBCT) (4, 10).

Both 3DCRT and IMRT are acceptable methods to provide HFRT (3–5). However, several considerations should be made when choosing one over the other (**Table 2**). When delivering 3DCRT, portal images or similar must be used to verify monitor unit (MU) output for each beam in a treatment plan (11). For IMRT treatments, the complex dose distributions with steep gradients near critical structures make portal images unreliable for validating OAR avoidance (12). To validate IMRT plans, the cumulative dose distribution must be verified by patient-specific QA by phantom dosimetry measurements (12).

**Table 2:** A summary of prostate cancer HFRT technical requirements.

Modality	Infrastructure Requirements	Personnel Requirements	QA Requirements
3DCRT	CT simulator (11) Immobilization (4, 10) Water Phantom (11) Ion Chamber (11)	Oncologist (11) Physicist (11) Dosimetrist (11) Therapist (11)	Safety Devices (11) Alignment Systems (11) CT & LINAC Mechanical Checks (11) CT Sim Image Quality (11) LINAC MU Output/Beam Quality (11)
IMRT	CT simulator (11) Immobilization (4, 10) Direct Dose Monitor (12) Point Dosimeters (12) Film/Detector Arrays (12) IMRT Phantoms (12) EPID (12)	Oncologist (11) Physicist (11, 12) Dosimetrist (11, 12) Therapist (11)	Safety Devices (11) Alignment Systems (11) CT & LINAC Mechanical Checks (11) CT Sim Image Quality (11) LINAC Direct Output/Beam Quality (12) Patient-Specific QA (12) Dose Monitor Performance (12) Transit Dosimetry (12)
IGRT	CT simulator (11) Immobilization (4, 10) Fiducials (10, 13) 4DCT/Gating (10, 13, 14) Motion Tracking (10, 14)	Oncologist (11) Dosimetrist (11, 12) Physicist (11–13) SBRT-Trained Therapist (10, 11, 13)	Safety Devices (11) Alignment Systems (11) CT & LINAC Mechanical Checks (11) CT Sim Image Quality (11) LINAC MU Output/Beam Quality (11) Imaging Dose (13) Patient Alignment (13) Prostate Position Verification (10) Surrogate-Motion Correlation (10, 13, 14) Image Quality/Operation (13)

#### Image-Guided Radiotherapy

Previous trials demonstrate HFRT effectiveness using varying levels of image guiding techniques (3–5). Although not required, IGRT can provide even greater accuracy for RT QA. For hypofractionated RT, IGRT is universally recommended to deliver more safe and effective treatment (6). Additionally, the use of supine or prone positioning body frames and fiducial markers are highly recommended but are not to be the sole technique to address patient alignment and motion management for Ultra-HFRT or SBRT techniques (**Table 1**) (4, 10). TGR 101 recommends the use of fiducial markers with either volumetric imaging by kV or MV CT or CBCT, or by multiple room-mounted 2D kV radiographs (10). Recommended motion management techniques are described in detail in the TGRs 101 and 76 (10, 14).

#### Quality Assurance

Regarding QA protocol, annual, monthly, and daily QA tasks are required to maintain a safe and accurate system eligible for high-dose delivery with participation of a qualified medical physicist. These tasks include, but are not limited to, CT and treatment room redundancy tests, and end-to-end detector and phantom tests (**Table 2**) (10). QA for HFRT should not stop at regular testing, but also occur during treatment. A qualified physicist should be present during the first fraction and readily available during all subsequent fractions of a HFRT course (10). Considering the compressed HFRT schedules, some weekly QA checks may need to be performed more frequently. The following tasks are recommended in TGR 100 (15):

•Confirm that the patient delivery script information or files are unchanged through the course of treatment, unless planned changes are implemented
•If changes are requested, confirm that they were correctly implemented, and are reasonable and justified to satisfy the overall prescription for the treatment
•Verify that all treatments are correctly documented and recorded comparison of dose to date with the prescription and planned end of treatment
•Review of treatment delivery system interlocks, overrides and problems, determination of the reason for these problems, and analysis of the need for corrections or other responses
•Review of recorded patient setup position, positioning shifts, image guidance decisions, and review of table position overrides and other indicators of shifted position

Based on the LINAC QA guidelines of TGR 40, clinics should consider performing the following tasks more frequently than standard when delivering HFRT, regardless of choice between 3DCRT, IMRT, or IGRT. On a daily basis, we recommend to verify the functionality of all safety mechanisms and interlocked accessories (blocks, compensators, bolus, etc.) (11). Additionally, mechanical checks (alignment indicators, gantry/collimator/couch position, etc.) and dosimetric checks (x-ray/electron output, beam flatness, etc.) should be tested for acceptable tolerance each day (11).

For treatment plan verification, 3DCRT planning requires the proper calibration and QA of the appropriate measurement devices (ion chambers, EPID, etc.) (11). IMRT planning, however, requires direct dose measurement to verify treatment plans effectively. Daily IMRT QA tasks require plan-specific verification using a phantom with point dosimeters or detector arrays, also requiring routine checks of the direct dose monitor and phantom detectors (12).

Applying methods of IGRT requires additional QA and considerations beyond those required by 3DCRT or IMRT. With the recommended implementation of implanted fiducial markers and patient immobilization devices, the prostate position must be verified before and during each fraction (4). The position verification will require daily performance checks on the imaging system for that task as well as image dose monitoring. Motion tracking techniques may be used if necessary, but require to be verified as a valid and accurate method of real-time localization (10, 13, 14). Prostate-surrogates (fiducial markers, skin markers, etc.) must have adequate motion correlation to the prostate to be effective (10, 13, 14).

## Discussion

These guidelines were designed with consideration of a LMIC clinic with access to the following resources: MV LINAC, on-board image guidance equipment, CT simulator, 3DCRT or IMRT TPS, and HFRT-trained faculty. Summaries of recommended treatment and QA guidelines are provided in **Tables 1** and **2**.

As of now, there is no standard HFRT practice documentation. HFRT is still developing in clinical trials for various cancer types and therefore has little data supporting a universal method of delivery. For this reason, these guidelines were compiled based on clinical trial parameters and recommendations from professional organizations. ASTRO, ASCO, and AUA collaborated to provide recommendations on prostate HFRT patient criteria and prescription doses and refer to the clinical trials for plan optimization parameters (6). To fill in gaps regarding HFRT-relevant basic-to-complex QA methods, we took conservative recommendations from various TGRs. The referenced TGRs include recommendations regarding stereotactic body RT practices, image guidance and motion management practices, LINAC performance, and patient-specific verification (10, 11, 13, 14, 16, 17).

When either moderate or ultrahypofractionated RT is undertaken, meticulous attention to the technical aspects of treatment planning, quality assurance and delivery are important. We recommend the general principle that to confidently replicate the results of published clinical trials and practice guidelines, the approach used in that study should be followed to the extent possible. The HFRT clinical trials have been done in predominantly White patient populations. We also recommend a need for implementation research HFRT trials that includes large population of Black patient populations e.g. in African LMICs. These recommendations are to be refined as more information and technological developments are presented in future investigations. Greater HFRT clinical trial participation by LMIC facilities would provide significant data to develop more HFRT-specific literature.

The evidence-based recommendations presented here are meant to be used as a practical guide to practice prostate cancer HFRT safely and effectively for LMIC facilities. We hope this guide can provide uniform guidance and improve comfortability for LMIC facilities to adopt HFRT to combat increasing cancer burden. The adoption of HFRT in LMIC facilities could significantly increase patient access to treatment at a fraction of the cost by reduced treatment schedules and faster-paced waiting lists. This guideline should not be deemed inclusive of all proper methods of care or exclusive of other methods reasonably directed to obtaining the same results. The radiation oncology health professionals (physician, medical physicists therapist) must make the ultimate judgment regarding any specific therapy in light of all circumstances presented by the patient. The authors assume no liability for the information, conclusions, and findings contained in these guidelines. These guidelines was prepared on the basis of information available at the time the authors conducted research, review and discussions on this topic. There may be new developments that are not reflected at this time and that may, over time, be a basis to revisit and update the guidelines, including after planned HFRT trials in Africa.

## Data Availability Statement

The original contributions presented in the study are included in the article/**Supplementary Material**. Further inquiries can be directed to the corresponding author.

## Author Contributions

Study design was contributed by WS, RS, WN, and LI. Data acquisition was contributed by WS, RS, and ML. Data analysis was contributed by WS, ML, WN, and LI. Manuscript writing was contributed by WS, RS, ML, AE, ES, WN, and LI. All authors contributed to the article and approved the submitted version.

## Funding

This work is partially supported by the Global Health Catalyst funding and National Institutes of Health under Award Number R01CA239042. The content is solely the responsibility of the authors and does not necessarily represent the views of the National Institutes of Health.

## Conflict of Interest

The authors declare that the research was conducted in the absence of any commercial or financial relationships that could be construed as a potential conflict of interest.

The Reviewer NL declared a past co-authorship with several of the authors WS, RS, AE, ES, WN, and LI to the handling editor.

## Publisher’s Note

All claims expressed in this article are solely those of the authors and do not necessarily represent those of their affiliated organizations, or those of the publisher, the editors and the reviewers. Any product that may be evaluated in this article, or claim that may be made by its manufacturer, is not guaranteed or endorsed by the publisher.
