# Supplementary material for: Practical Guidelines on Implementing Hypofractionated Radiotherapy for Prostate Cancer in Africa
Source: Front Oncol. 2021 Dec 1;11:725103. doi: 10.3389/fonc.2021.725103 (PMC8673781; doi:10.3389/fonc.2021.725103)
Supplement: Supplementary file 1 [file DataSheet_1.pdf]

Supplementary Material

Supplementary Figure 1. Global Cancer Observatory estimations on the total African prostate cancer burden, projected up to 2040, measured by annual incidences and mortalities.[2]

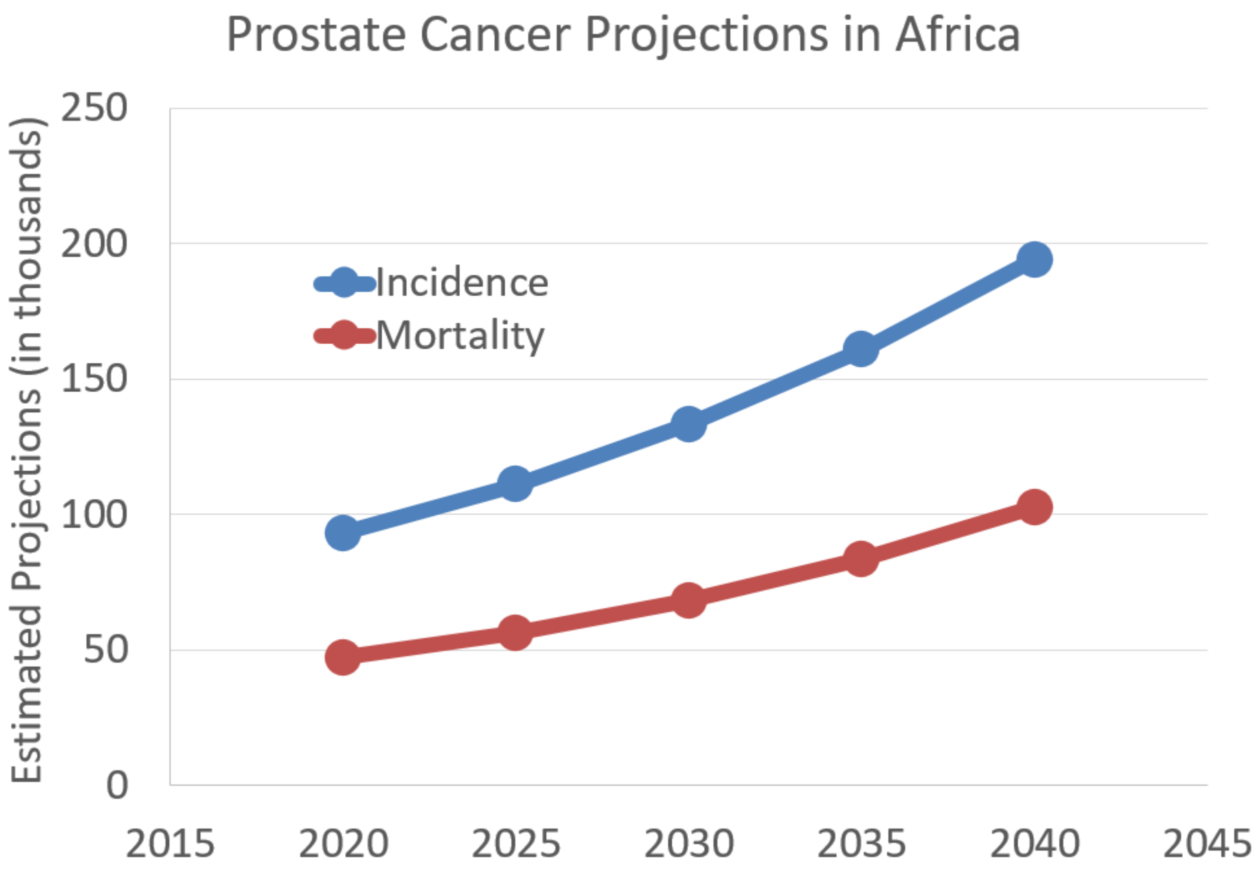

Supplementary Figure 2. (A) Number of radiotherapy centers in African countries as reported from the Directory of Radiotherapy Centers.[1] (B) Reported prostate cancer incidences in Africa during 2020 as reported by the Global Cancer Observatory.[2]

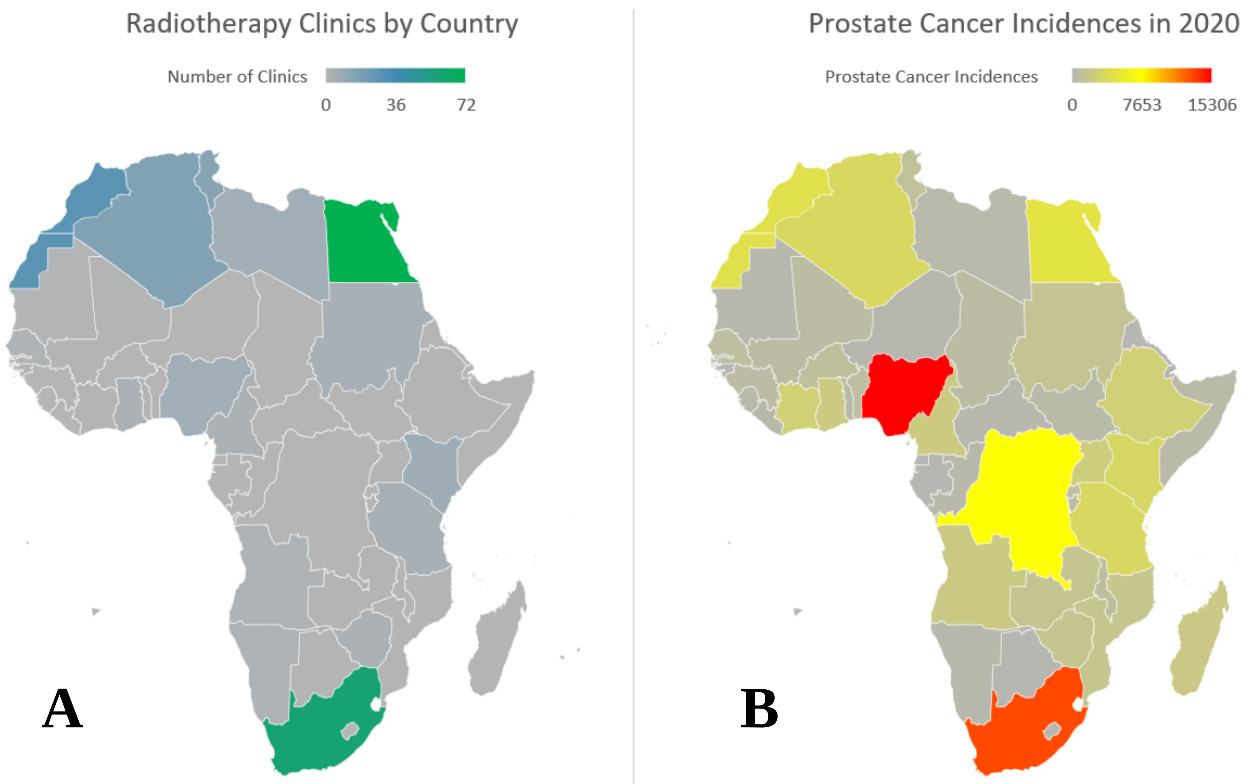

References

1. International Atomic Energy Agency: Division for Human Health: DIRAC (DIrectory of RAdiotherapy Centres) [updated 12/11/2018] [Internet]. 12/11/2018 , 2018[cited 2019 Oct 23] Available from: https://dirac.iaea.org/Query/Map2?mapId=2

2. International Agency for Research on Cancer: Global Cancer Observatory (GLOBOCAN) [Internet]. World Heal Organ 593:1–2, 2018[cited 2019 Jul 1] Available from: http://gco.iarc.fr/
